# The Impact of Financial Incentives on Service Engagement Among Adults Experiencing Homelessness and Mental Illness: A Pragmatic Trial Protocol

**DOI:** 10.3389/fpsyt.2021.722485

**Published:** 2021-08-03

**Authors:** Nadine Reid, Rosane Nisenbaum, Stephen W. Hwang, Anna Durbin, Nicole Kozloff, Ri Wang, Vicky Stergiopoulos

**Affiliations:** ^1^Centre for Addiction and Mental Health, Toronto, ON, Canada; ^2^MAP Centre for Urban Health Solutions Applied Health Research Centre, Li Ka Shing Knowledge Institute, St. Michael's Hospital, Unity Health Toronto, Toronto, ON, Canada; ^3^Division of Biostatistics, Dalla Lana School of Public Health University of Toronto, Toronto, ON, Canada; ^4^Department of Medicine, Faculty of Medicine, University of Toronto, Toronto, ON, Canada; ^5^Department of Psychiatry, University of Toronto, Toronto, ON, Canada; ^6^Institute of Health Policy, Management and Evaluation, University of Toronto, Toronto, ON, Canada

**Keywords:** homelessness, mental illness, service engagement, financial incentives, randomized controlled trial

## Abstract

**Background:** People experiencing homelessness and mental illness have poorer service engagement and health-related outcomes compared to the general population. Financial incentives have been associated with increased service engagement, but evidence of effectiveness is limited. This protocol evaluates the acceptability and impact of financial incentives on service engagement among adults experiencing homelessness and mental illness in Toronto, Canada.

**Methods:** This study protocol uses a pragmatic field trial design and mixed methods (ClinicalTrials.gov Identifier: NCT03770221). Study participants were recruited from a brief multidisciplinary case management program for adults experiencing homelessness and mental illness following hospital discharge, and were randomly assigned to usual care or a financial incentives arm offering $20 for each week they attended meetings with a program provider. The primary outcome of effectiveness is service engagement, measured by the count of participant-provider health-care contacts over the 6-month period post-randomization. Secondary health, health service use, quality of life, and housing outcomes were measured at baseline and at 6-month follow-up. Quantitative data will be analyzed using descriptive statistics and inferential modeling including Poisson regression and generalized estimating equations. A subset of study participants and other key informants participated in interviews, and program staff in focus groups, to explore experiences with and perspectives regarding financial incentives. Qualitative data will be rigorously coded and thematically analyzed.

**Conclusions:** Findings from this study will contribute high quality evidence to an underdeveloped literature base on the effectiveness and acceptability of financial incentives to improve service engagement and health-related outcomes among adults experiencing homelessness and mental illness.

## Introduction

People experiencing homelessness and mental illness have significantly poorer mental and physical health and quality of life relative to the general population (1–3). In addition, compared to the general population, they have higher rates of acute care utilization and associated costs (2, 4, 5). Efforts to improve the health and health service use outcomes of this population have historically been challenged by poor service engagement and high drop-out rates (6). Lack of service engagement has in turn been associated with poor health outcomes, including increased symptom severity, reduced quality of life, a greater likelihood of returning to hospital (6–8), and increased acute care costs (6, 7). Strategies to improve service engagement are essential to maximizing treatment effectiveness and to improving the health and social outcomes of this population.

For adults experiencing homelessness and mental illness, the transition from hospital to community settings has been associated with an increased risk of homelessness (9) and worsened mental illness (10). Programs and interventions aiming to support this population in establishing (re)connections to housing and community-based resources during care transition, such as Critical Time Intervention, have been used successfully to improve the health (11), health service use (12–14), quality of life (11, 14), and housing outcomes (9, 15) of this population. Further research, focused on practical strategies to improve service engagement, particularly in the critical period following hospital discharge, is essential to maximizing the impact of such interventions and to improving health and social outcomes in this population.

An individual's decision to engage in and adhere to treatment is influenced by a wide range of factors. Behavioral economics principles in health care suggest that individuals have a tendency to make health decisions that are biased toward the present and immediate rewards vs. future outcomes and delayed gratification (16, 17). These principles have been used in intervention design to motivate individuals to make health-promoting decisions, including engaging in treatment or services (18). Literature on interventions to promote service engagement among people experiencing mental illness has suggested that the provision of practical housing and financial supports are important in promoting service engagement (19, 20). It has also been proposed that financial incentives, providing a reliable, practical, and immediate reward for health-promoting behaviors, may be an effective engagement strategy and positively influence health decision-making, and consequently improve health and health service use outcomes in vulnerable populations (18, 21–24).

Financial incentives have indeed been shown to influence health behaviors for a range of health conditions, including increasing smoking cessation rates (25, 26), weight loss (27), engagement in HIV care and prevention (28–30), blood donation (31), and adherence to tuberculosis treatment (32). Reviews of the literature have found that financial incentives were effective in 73% of cases (33), and that effects were greater for singular behaviors compared to complex, sustained behavior change (33) and for shorter-term (<6 months) interventions (22). Specifically among people experiencing homelessness and/or mental illness, financial incentives have been shown to improve health outcomes such as therapy adherence (34, 35), medication adherence (32, 36), substance use abstinence (37, 38), and smoking cessation (39, 40).

Although existing literature has highlighted that financial incentives may be an effective service engagement strategy for underserved populations, particularly when implemented in the context of a short-term intervention (22, 33, 38), significant knowledge gaps remain. In particular, there is limited evidence on the effectiveness of financial incentives in different populations and settings, including their use in adults experiencing homelessness and mental illness following hospital discharge, a period of high risk for poor health outcomes. In addition, few studies have explored the acceptability of financial incentives, including the perspectives of key stakeholders, such as affected individuals and their service providers. As the use of financial incentives remains controversial, and ethical and pragmatic concerns continue, including concerns about coercion and unintended consequences, further exploration of these issues is warranted (41–44).

To address these knowledge gaps, this article describes an evaluation protocol for a study using mixed methods to investigate the effectiveness of and experiences with using financial incentives to increase engagement of homeless adults with mental illness with a brief case management intervention following hospital discharge in Toronto, Canada. In addition to service engagement, using a pragmatic field trial design, this study will investigate the impact of financial incentives on secondary health, health service use, quality of life, and housing outcomes. Qualitative data, exploring the acceptability, and perceived positive and negative impacts of financial incentives, will be integrated in study findings to support a comprehensive and nuanced understanding of the potential role of financial incentives in supporting service engagement in this underserved population.

## Methods and Analysis

### Program Description

The Coordinated Access to Care for the Homeless (CATCH) program is a multidisciplinary brief case management program for individuals experiencing homelessness and mental illness being discharged from hospital in Toronto, Canada. Informed by the Critical Time Intervention model, the program was launched in 2010 and has been described extensively elsewhere (11, 12, 45–48). Briefly, CATCH aims to support individuals in the critical period following hospital discharge by providing coordinated physical and mental medical care, peer support and intensive case management over a period of 3–6 months. Previous program evaluations have associated CATCH with improvements in mental health and substance use symptoms and health status (11), fewer and shorter hospitalizations, more frequent outpatient psychiatrist visits (46), and continuity of care (45).

### Study Design

Using a community-based, participatory research framework, CATCH-Financial Incentives (CATCH-FI) is a pragmatic field trial using mixed methods to evaluate the impact and acceptability of financial incentives in promoting service engagement of homeless adults with mental illness following hospital discharge. This study was launched in December 2018. Study recruitment is completed, data collection however is ongoing and the trial is registered with ClinicalTrials.gov (Identifier: NCT03770221).

CATCH program participants enrolled in the CATCH-FI trial and randomly assigned to the intervention arm receive $20 for every week they remain meaningfully engaged with program service providers over 6 months of follow-up, or until they are successfully transitioned to longer-term supports. Study participants can earn up to $80CAN per month by attending meetings with their program service provider by phone, text, email, or in-person, as per their care plan. Participants randomly assigned to the control arm receive usual CATCH care, which does not include a financial incentive for attending meetings with their program service provider.

The impact of financial incentives on participants' level of service engagement, measured by program attendance, evaluated as the number of “health-care contacts” a participant makes with CATCH service providers over a 6-month follow-up period. Of note, participant contacts with service providers are counted as “health-care contacts” if they relate to participants' care plans. As a low barrier program, CATCH service providers meet program participants in person, via phone, email, or texts, as per program participants' needs and preferences. Social or trivial contacts of program participants with service providers are not considered “health-care contacts” and are not being measured or documented in program records or reports to program funders.

Secondary health, health service use, quality of life, and housing outcomes (secondary outcomes) are also being collected over the study period. The study hypothesis is that participants receiving financial incentives will have higher levels of service engagement and consequently better health, health service use, quality of life, and housing outcomes compared to participants receiving usual care.

This study additionally uses qualitative methods to investigate stakeholder perspectives and experiences using financial incentives. In-depth qualitative interviews and focus groups were conducted with study participants, program service providers, and other key informants to explore experiences of and perspectives on the acceptability of using financial incentives to support service engagement in this population.

The research questions guiding this study are:

1. What are the levels of service engagement and health, health service use, quality of life, and housing outcomes for homeless adults with mental health needs receiving financial incentives vs. usual care over a 6-month period following hospital discharge?
2. What are key stakeholder perspectives and experiences using financial incentives, including their acceptability, feasibility, as well as potential drawbacks?

#### Approach to Mixed Methods and Data Integration

Within a community-based, participatory research framework, a convergent mixed methods design is used to evaluate experiences with, perspectives on, and impact of financial incentives on service engagement of an underserved population. With the qualitative sample drawn from the larger study sample, and inclusive of additional key stakeholders, qualitative, and quantitative data collection take place in parallel (49, 50). In addition to integrating qualitative and quantitative data sources through sampling, integration of data sources will take place by comparing and contrasting qualitative and quantitative data once data analysis is completed, and through interpretation and reporting of findings (50–53). Our objective in employing this convergent synthesis approach is to leverage the respective advantages of both types of data to generate a comprehensive and nuanced understanding on how financial incentives may facilitate engagement, health, and well-being of an underserved population, including the range of unique, multi-level contextual factors that may impact implementation of this strategy (50, 54).

### Study Participants: Recruitment, Eligibility, and Randomization

The CATCH program receives 450–600 referrals of homeless adults per year, from hospitals or community agencies, prior to or shortly after hospital discharge for a mental health condition. Study participants were recruited among successive new CATCH program participants. Referrals to the study were made by CATCH staff during program intake meetings. Program participants expressing an interest in receiving information about the study were contacted by research staff to confirm interest, eligibility, to obtain informed consent, and to conduct a baseline assessment.

Study participants meet both CATCH program and CATCH-FI study eligibility criteria. Program eligibility criteria include: (1) current homelessness (defined as having no fixed place to stay for at least the past seven nights with little likelihood of finding a place in the upcoming month) or precarious housing (defined as currently occupying a single room in a multi-tenant building or house with shared common areas including bathroom and kitchen or a hotel/motel as a primary residence, and having a history of one or more episodes of absolute homelessness in the past year); (2) service provider-determined unmet mental health needs; (3) service user-determined unmet support needs; and (4) age 18 years or older. Excluded from the program are individuals with recent aggressive behavior requiring a higher intensity of support, or individuals whose illness severity necessitates residential care. To be eligible for the current study, in addition to meeting program eligibility requirements, participants must have been new referrals to CATCH, recently admitted or readmitted to hospital services, and have had at least one contact with the CATCH team.

Participants were randomized following the baseline interview using block randomization (55), which randomizes the participant list into groups 1:1 using blocks of four and six. This approach has the advantage of maintaining balanced group sizes throughout the recruitment process. The nature of the intervention precluded blinding of participants and study staff.

A subset of participants completing qualitative interviews were purposefully recruited from the larger randomized sample to participate in in-depth semi-structured qualitative interviews. Qualitative study participants were purposefully selected to reflect a diversity of perspectives based on gender, ethnicity, study arm, and service engagement. Study staff invited individuals with the ability to reflect on their experiences, a strategy that has previously been used with success by our team in studies of adults experiencing homelessness and mental illness (11, 12, 45–48). Other key informant stakeholders and CATCH service providers were also purposefully recruited to complete interviews and focus groups based on their role or expertise (health care, health administration, health ethics). Key informants were health and administrative leaders at organizations affiliated with the CATCH program, and had experience serving the target population. CATCH service providers included both case managers and program administrators.

### Sample Size

Previous research by our group (48) estimated that for those receiving usual care, the mean number of health-care contacts with program providers per month is 4.6. It was hypothesized that the intervention group will have at least 25% more health-care contacts with program providers per month (mean = 5.8) during the critical transition period. Because the primary outcome represents a count variable, sample sizes for Poisson regression were calculated using the formula provided in Signorini (56). Sample sizes of 67 per group achieve 80% power to detect a rate ratio of 1.25 with a significance level of 0.05 using a two-sided test. However, we expect an attrition rate of 22.0% (44), thus the final sample size per group is inflated by 22%, resulting in a total of 172 study participants. A subsample of study participants (*n* = 22), service providers (*n* = 12), and other key informants (*n* = 6) was estimated a priori as adequate to achieve saturation of qualitative findings and triangulation of data sources.

### Data Collection

Baseline data collection occurred between November 2018 and September 2020; follow-up data collection will be completed in July 2021. All data collection is conducted by trained research assistants from the Survey Research Unit at the Centre for Urban Health Solutions at St. Michael's Hospital. Quantitative surveys lasting 1–2 hwere administered at baseline (up to within 6 weeks of enrollment) and are offered at 6 months post-enrollment (between 6 weeks prior to and up to 16 weeks after that) to all study participants. Survey data are collected using SNAP professional software, and data are held on an internally owned and operated secure sever. All program participants received honoraria paid in-person, by check or email money transfer for each completed interview ($30 for the baseline interview and $60 for the 6-month follow-up interview), in addition to public transportation fare.

Qualitative data collection occurred between April 2019 and December 2020. In-depth, 45–60-min semi-structured interviews were conducted during the study period with program participants and other key informants, and focus groups were conducted with CATCH service providers. Qualitative interview service user participants received an honorarium of $30 and public transportation fare.

This study uses several evidence-based follow-up and study retention strategies for this population (57, 58). To minimize attrition, participants were asked at baseline to provide detailed contact information for themselves and any family, friends, or service providers who could help locate them. To support study retention further, participants were also encouraged to call study staff monthly to update contact information (receiving a $10 honorarium per call). In addition, study staff regularly reach out to participants between interviews to maintain up to date contact records.

**Figure 1:**
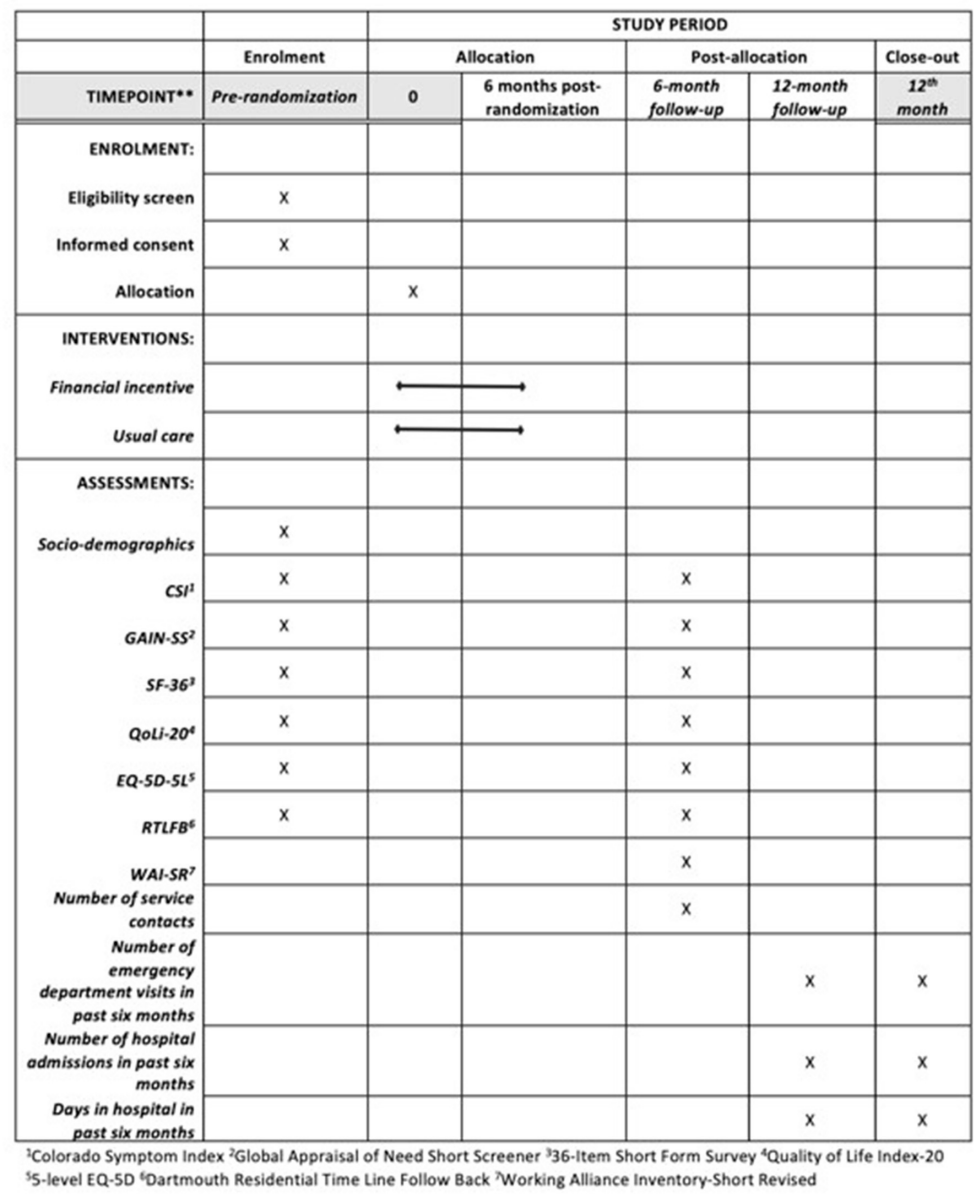
Schedule of enrolment, intervention, and assessments.

A schedule of enrolment, intervention, and assessment is detailed in Figure 1.

### Measures

#### Quantitative Measures

The primary outcome is service engagement, or program attendance, measured as a count of health-care contacts with CATCH service providers per month over the 6-month period (or until discharge from the program). CATCH service providers record program participant attendance in health care appointments and care planning meetings lasting at least 5 min in participant health records. Eligible health-care contacts include in-person and virtual appointments, as well as care planning conversations through email or text. This definition is consistent with the current program practices and ensures only eligible health-care contacts are recorded and reported to the funding agency. Data on program attendance will be captured at study end through chart reviews by a blinded study staff.

Demographic data and other participant characteristics including residential status and income sources were collected by self-report at baseline. Secondary outcome measures of mental and physical health status, health service use, quality of life, and housing are being collected at baseline and 6 months and a measure of perceived therapeutic working alliance is collected at 6 months. Health service use will also be evaluated using data linkage of administrative health records, conducted at ICES, which holds population-based health and health service use information at the patient level for all Ontarians with health insurance.

See Table 1 for a detailed description of all quantitative measures.

**Table 1:** Description of measures.

**Domain**	**Instrument**	**Description of instrument**
**Service engagement**		
	Chart review	Number of contacts with CATCH service providers per month over 6 months or until discharge from the program. Contacts are calculated by considering the number of appointments attended, phone calls, texts, emails, no-shows, and cancellations and drop-outs. Synchronous interactions must last at least 5 min to be counted as meaningful, consistent with funder reporting requirements.
**Demographics**		
	Self-report	Self-reported age, gender, ethnic or cultural identity, country of birth, main language, education level, marital status, residential status (duration of homelessness during lifetime and past 6 months), income sources in past 30 days for self and partner (if applicable), including all jobs, Ontario Works, Ontario Disability Support Program, Employment Insurance, Child Benefits, and Child Support
**Health and mental health status**		
	Colorado Symptom Index (CSI)	The CSI (59) measures mental health symptom severity and was specifically designed for and has been widely used among individuals experiencing homelessness and mental health problems. It includes 14 items rated on a five-point scale assessing the presence and frequency of symptoms of mental illness experienced in the past 30 days. Evidence of internal consistency (0.92), test-retest reliability (0.71), and validity is strong and scores are significantly correlated with functioning.
	Global Appraisal of Individual Needs-Short Screener (GAIN-SS)	This screener version of the GAIN (60) has been used extensively in homeless populations with mental health problems to evaluate the severity of substance use problems. It includes a series of questions regarding substance use problems like getting into fights, problems at work, and withdrawal symptoms in recent months. A past month score reflects the frequency with which participants identified past month problems. Other outcomes of interest include the number of days in the past month that participants report problems with substance use; and the amount of money spent on alcohol or drugs in the past month.
	36-Item Short Form Survey (SF-36)	The SF-36 (61, 62) is a widely used measure of generic health status with excellent psychometric properties that has been used successfully in a variety of settings and diagnostic groups, including homeless populations (63). Items span eight domains of health including general health perceptions, vitality, bodily pain, general mental health and limitations in physical, social and usual role activities because of physical or emotional health problems. Scoring yields a Physical Component Summary (PCS) score and a Mental Component Summary (MCS) score.
**Health service use**		
	Self-report	Self-reported number of emergency department visits, hospital admissions, and days in hospital in the past 6 months for pre- and post-randomization.
	ICES administrative data linkage^a^	Number of emergency department visits, hospital admissions, and physician visits in the 12 months pre- and post-randomization.
**Quality of life**		
	Quality of Life Index-20 (QoLi-20)	This is a validated shorter version of the Lehman Quality of Life Scale (64) that has been used successfully in the homeless population (65–67). It consists of 20 items across seven subjective scales (reflecting living situation, everyday activities, family, social relationships, finances, safety, and satisfaction with life in general) and four objective scales (reflecting everyday activities, having enough money, family contacts, and contacts with friends).
	5-Level EQ-5D (EQ-5D-5L) and EQ visual analog scale (EQ VAS)	The EQ-5D-5L (68) is the 5-level version of the EQ-5D, a widely used measure of generic health-related quality of life that has been used in populations with mental illness (69). This version asks participants to rate perceived problems on a five-point Likert scale across five dimensions: mobility, self-care, usual activities, pain/discomfort, and anxiety/depression. Scoring is weighted and yields a single utility score between zero and one that describes the user's health state. The EQ VAS asks participants to rate their health on a vertical visual analog scale with opposite ends labeled “The best health you can imagine” and “the worst health you can imagine.” Participants' visual ratings are then assigned to a quantitative value ranging from zero to 100.
**Housing stability**		
	Dartmouth Residential Time-line Follow-Back (RTLFB)	This modified version of the RTLFB (70, 71) has successfully been used in past studies of homeless populations (72, 73). It uses a calendar and prompts to collect detailed information about participants' type of housing and number of days stably housed for specific time periods. A variable representing time spent homeless in the past 6 months in number of days is included as a baseline demographic measure of homelessness. The primary housing outcome of interest from this scale is the number of days stably housed in the past 6 months.
**Working alliance**		
	Working Alliance Inventory-Short Revised (WAI-SR)	This shorter version of the Working Alliance Inventory (74, 75) assesses participant-perceived therapeutic relationship with a case manager. It includes three subscales—task, goal and bond—with items rated on a five-point Likert scale. Scoring yields a single summary score ranging from 12 to 60 with higher scores indicating a stronger therapeutic relationship.

a *Databases accessed and linked by ICES include the National Ambulatory Reporting System, Discharge Abstract Database, Ontario Mental Health Reporting System, and Ontario Health Insurance Plan*.

#### Qualitative Measures

Interviews and focus groups explored the experiences of using financial incentives from both program participant and provider perspectives. Topic guides were developed and iteratively refined by the PI and study staff, with input from people with lived experience of homelessness and mental health challenges. Topics included perceptions of facilitators and barriers to service engagement during care transitions; factors affecting health decision-making in this population; and the perceived risks, barriers, and expected or experienced impact of financial incentives during care transitions. Given ethical concerns and underdeveloped literature on the use of financial incentives, topic guides specifically probed stakeholders to comment on the acceptability of using financial incentives, facilitators of ethical implementation, and potential negative or unintended consequences.

Interviewers' extensive experience with the study population, rigorous interviewer training for this study, and early and ongoing review of transcripts by the PI and study staff helped to ensure consistency across interviews. Investigator triangulation and member checking will help to validate the findings.

### Analyses

#### Quantitative Analyses

Exploratory analyses will calculate descriptive statistics (mean, standard deviation, median, quartiles), construct graphs (histograms, box-plots, scatterplots, spaghetti plots), and estimate correlations between selected participants' characteristics and longitudinal outcomes.

##### Primary Outcome Analysis

Since program duration is customized for each participant, and may last between 1 and 6 months, we will calculate participants' person-months of program participation. This will allow us to estimate the rate ratio comparing the intervention and usual care groups with respect to the number of contacts with CATCH service providers per month. Therefore, for each participant, the total number of months in the program before discharge and the total number of contacts over the number of months in the program will be calculated. A Poisson regression model (PROC GENMOD) with total contacts as the dependent variable, group (CATCH-FI vs. CATCH-UC) as the covariate and an offset equal to the log (number of months spent on the program) will estimate the rate ratio and 95% confidence intervals, and the mean number of contacts per person-months and 95% confidence intervals in each group.

##### Secondary Outcome Analysis

For continuous outcomes (i.e., QoLi-20, CSI, SF-36, and EQ-5D VAS), we will define change from baseline to 6-month follow-up as scores at 6 months minus scores at baseline. We will conduct analyses of covariance (ANCOVA) to compare changes from baseline between CATCH-FI and CATCH-UC, adjusting for baseline scores as a covariate.

For count outcomes (i.e., GAIN-SS, number of hospitalizations, number of days hospitalized, and number of emergency department visits) we will model the baseline and 6-month outcomes using generalized estimating equations (GEE) assuming the Poisson distribution or the negative binomial distribution, if over-dispersion is suggested by the data. The models will include the main effects of group (CATCH-FI vs. CATCH-UC) and time (6 months vs. baseline), and the interaction of group by time. A significant interaction will indicate that change from baseline is different between the groups. Rate ratios and 95% confidence intervals will be estimated.

The analysis of administrative data is similar to that of count outcomes, except that the period of consideration will be 12 months instead of 6 months pre and post-randomization.

For analyzing the number of days stably housed in the past 6 months, we will consider GEE with a Poisson or negative binomial distribution. The model will include the main effects of group and time, an interaction between group and time, selected covariates, and an offset represented by the natural log of residence days accounted during the 6 months interval. Rate ratios and 95% confidence intervals will be estimated to compare the rate of days stably housed per person-months.

For the WAI-SR, evaluated at the 6-month follow-up only, total scale, and sub-scales scores will be calculated and compared between the groups using the two-sample *t*-test or the Wilcoxon rank-sum test if extreme outliers are present. The correlation between WAI-SR and other outcomes at 6 months will be explored by estimating the Pearson or Spearman correlation coefficients, overall and by group.

SAS 9.4 will be used for all analyses and all analyses will use the intention-to-treat principle. We will consider multiple imputation to handle missing data. All statistical tests will be two-sided and a *p*-value of 0.05 or less will indicate statistical significance. There are no plans for interim analyses.

#### Qualitative Analyses

All interviews and focus groups were audio-recorded and transcribed verbatim. Grounded theory (76, 77) and inductive thematic analysis (78) will be used to guide coding and interpretation of interview and focus group transcripts. This approach allows for theory-testing based on existing literature in addition to anticipating novel emerging themes.

Coding will be completed by a team of coders including the PI, study co-Investigators, and study staff, using a structured approach to maximize rigor (79–81). First, transcripts will be collectively reviewed to develop a set of key concepts or codes; potential codes are both identified beforehand based on literature reviews and initial data impressions and allowed to emerge during coding. Similar codes will be grouped into sets of higher-order themes, supported with direct examples, and the research team will reconvene to discuss categories and collectively, iteratively reduce, and refine the set of themes as needed. Investigator triangulation and member checking will be used to validate the data. Qualitative data analysis software (QSR International NVivo 12) will be used to support all qualitative data management and analysis.

### Benefits, Risks, Safety, and Monitoring

All study participants have access to the CATCH program throughout the trial period. Participants in the intervention group experience the direct benefit of receiving a financial incentive. Participants in both groups may indirectly benefit from sharing their experiences with study staff and by contributing to knowledge creation that may inform strategies to more effectively support this population. Involvement in this intervention poses minimal risk to the safety of participants, and no anticipated harms.

A key criticism of the use of financial incentives is the risk of coercion. The study strives to minimize this risk directly, by recruiting participants from a program providing comprehensive support to homeless adults with mental health challenges, irrespective of study participation. In addition, the study uses a modest financial incentive and a rigorous informed consent process. Furthermore, the study strives to minimize the risk of coercion indirectly, by aiming to better understand this potential risk, how to minimize it in practice and how to identify strategies to better engage this underserved population.

It is possible that some participants may find certain survey or interview questions uncomfortable. Participation however is voluntary and individuals may choose not to answer or withdraw from the study at any point in time without penalty. All interventions involving financial incentives include the risk of creating a differential effect on those with varying levels of financial need, but this study's exclusive focus on people experiencing homelessness and mental health challenges minimizes this risk.

The study protocol was registered with ClinicalTrials.gov on December 10, 2018 (NCT number: NCT03770221). Research Ethics Board (REB)-approved protocol amendments will be posted on the site. The PI and study team will meet regularly to review data, data confidentiality, any adverse events, adherence to protocol design, recruitment and retention. In addition, the study team meet will meet regularly throughout the trial period, and collect and report to the REB any reported adverse events or other unintended effects of the intervention as per institutional policies. Important protocol modifications will also be reported to the REB and trial registry. This pragmatic field trial is subject to audits by the host institution.

The PI and study team bring extensive experience in the design, implementation, and evaluation of interventions for the target population (11, 12, 45–48), providing a reliable foundation for early identification and prompt response to potential emergent challenges.

## Discussion

This article describes a pragmatic field trial protocol aiming to evaluate the acceptability and impact of financial incentives on service engagement of homeless adults with mental health challenges following hospital discharge. Service engagement of this population in traditional health services remains low, given their multiple competing priorities of securing shelter, basic income, access to health and social services, and other needed supports. A pragmatic randomized field trial and in-depth qualitative interviews and focus groups will contribute high quality evidence to an underdeveloped literature on the effectiveness and acceptability of financial incentives in supporting service engagement of this population, at high risk of poor outcomes.

Using a participatory framework, the study aims to include the voices of all relevant stakeholders in data collection, analysis and interpretation, including affected individuals, direct service providers, program administrators, and other key informants. The protocol is strengthened by the use of mixed methods, to provide a nuanced understanding of the acceptability, risks, and impact of financial incentives, including ethical and pragmatic considerations associated with their use. Ultimately, the study aims to inform local health solutions to supporting service engagement of this population.

Results and lessons learned will be useful to other populations or jurisdictions interested in implementing financial incentives or seeking to improve service engagement of underserved populations, a priority in many settings aspiring to promote health equity. Future research should investigate the role of additional strategies to promoting service engagement, including flexible drop-in appointments, using peers, and proactive outreach, in efforts to understand what service engagement strategies work best, for who, in diverse service contexts.

## Conclusion

Promoting service engagement of homeless adults with mental illness following hospital discharge is urgently needed. Study findings will contribute to growing literature on strategies to support service engagement and improve health outcomes among disadvantaged and underserved populations.

## Ethics and Dissemination

### Ethics Statement

This study protocol was approved by the Unity Health Toronto Research Ethics Board (approval number REB#18-196; approved on November 1, 2018) and the Centre for Addiction and Mental Health Research Ethics Board (approval number REB#156/2018; approved December 19, 2018). All participants provided either written or verbal informed consent to participate. To facilitate and confirm participants' understanding, access to a professional interpreter and a capacity-to-consent questionnaire were used as needed.

### Dissemination and Data Sharing

Trial findings will be communicated to study participants, funders and research audiences through briefing notes, presentations, and publications. There are no publication restrictions. Authorship membership is limited to study co-investigators and ICJME criteria for authorship will apply. Study datasets will be available through the corresponding author, as per Unity Health Institutional policies.

## Author Contributions

NR led drafting of this manuscript. VS is the study's Principal Investigator and supervised the drafting of this manuscript. RN participated in the study design and implementation, led data analysis, and participated in the editing of this manuscript. SH, AD, and NK participated in study design and implementation and the editing of this manuscript. RW participated in data analysis and editing of this manuscript. All authors contributed to the article and approved the submitted version.

## Conflict of Interest

The authors declare that the research was conducted in the absence of any commercial or financial relationships that could be construed as a potential conflict of interest.

## Publisher's Note

All claims expressed in this article are solely those of the authors and do not necessarily represent those of their affiliated organizations, or those of the publisher, the editors and the reviewers. Any product that may be evaluated in this article, or claim that may be made by its manufacturer, is not guaranteed or endorsed by the publisher.
