# Supplementary material for: The Impact of Financial Incentives on Service Engagement Among Adults Experiencing Homelessness and Mental Illness: A Pragmatic Trial Protocol
Source: Front Psychiatry. 2021 Aug 3;12:722485. doi: 10.3389/fpsyt.2021.722485 (PMC8369574; doi:10.3389/fpsyt.2021.722485)
Supplement: Supplementary file 1 [file Data_Sheet_1.docx]

**Appendix A: Protocol**

**Coordinating Access to Care for People Experiencing Homelessness:**

**The role and impact of financial incentives (CATCH-FI)**

**Abstract**

Research on the use of financial incentives to increase service engagement has been promising, but many important questions remain regarding their use with different populations and types of services. This research aims to evaluate the effect of financial incentives in facilitating engagement with a brief case management intervention for people experiencing homelessness and mental illness after discharge from hospital. This study builds on prior research by our team on improving health outcomes and acute care utilization in this population at critical times of care transition. The study will examine the health, health service use, and housing outcomes for those receiving financial incentives compared to those receiving usual services over a 6 month period after hospital discharge. The study also aims to examine the experience of using financial incentives in this context from both participant and health system perspectives, to better understand the implications and challenges associated with the use of financial incentives in this population.

**Background and Rationale**

Poor engagement with services and low levels of treatment adherence remain a significant problem within mental health care. The cost of poor engagement and treatment non-adherence has several implications for both individuals (relapse, re-hospitalization, increased risk for adverse health outcomes and poorer subjective quality of life) and communities (increased healthcare costs, health disparities) (1, 2). Behavioral economics principles suggest that decision making in health is strongly influenced by contextual factors, beliefs, competing demands, emotions and other social-cognitive factors (3). Based on these principles, it has been hypothesized that financial incentives could influence health decision making by providing a certain and near-immediate reward for health-promoting behaviors (4, 5), and potentially offsetting the costs associated with seeking health care, such as travel or missed work (6). It has been therefore suggested that offering financial incentives to patients for reaching health goals may improve health outcomes (7).

Effective financial incentives employ basic principles of behavior reinforcement, including identification of target behavior, frequent collection of an objective measure of that behavior, selection of desirable reinforcement, and a consistent and immediate link between target behavior and re-enforcers (8). Conditional incentive-based approaches have been used successfully in multiple settings to promote healthy behaviors, including smoking cessation, weight loss, adherence to childhood vaccination, and antenatal clinic visits (9-13). A recent review of the health behavior literature showed that economic incentives worked 73 percent of the time, with stronger effects found for simple one-time behaviors than for complex behaviors requiring sustained effort (14). A further systematic review and meta-analysis also found that financial incentives are more effective than usual care in encouraging healthy behavior change, noting a greater effect in short term (<6 month) incentive interventions than in those longer than 6 months (5).

In the field of mental health, financial incentives have been considered as a possible motivating tool in improving therapy adherence and attendance, in promoting medication adherence, and in reducing illicit substance use. A study promoting psychotherapy attendance and adherence led to improved clinical functioning when monetary incentives were provided. This was a novel application of financial incentives -a progressively lowered pay scale (discounted fee) that rewarded therapy attendance and adherence (15). Attendance at therapy sessions was also shown to significantly increase among African-American patients with depression from 79% prior to receiving an incentive, to 86% during the intervention phase (i.e. incentive payments), with a subsequent significant decrease to 69% following discontinuation of incentives(16). Studies have also shown that financial rewards that are contingent on health-promoting behaviors can help people reduce substance use (17). In a study focusing on substance abuse, it was demonstrated that abstinence from cocaine remained significantly higher at 52 week follow up, 71-80% in those in the contingency management (CM) group vs 20% in the control group (18). Furthermore, meta-analyses of interventions involving the provision of vouchers contingent on abstinence and related behaviors have shown improved outcomes with an overall medium effect size (19, 20). These effects were larger the greater the voucher’s monetary value, and the closer in time they were given to the measurement of the targeted behavior (19).

An earlier systematic review undertaken to identify whether financial or material incentives improve treatment adherence in people with severe mental illness (SMI) concluded that financial incentives may improve treatment adherence in this population, during the period of time in which the incentive is offered (21). A subsequent cluster randomized controlled trial of patients with psychotic disorders who did not adhere to treatment demonstrated that offering financial incentives improved adherence to antipsychotic depot medication over a 1-year period (22, 23), with adherence returning to approximately baseline level once incentives were discontinued (24).

UK based insurance companies, Medicare and physician funding plans in Canada have initiated pay-for-performance (P4P) programs rewarding health providers for achieving certain evidence-based performance goals. Despite an input of $2.8 billion from the UK government, health outcomes did not improve over a 7-year period (25). Determinants of P4P failure were many in that context, but one major cause was the limited understanding of the complexities of effecting behavior change. Directing P4P efforts towards rewards for patients achieving evidence-based health goals, rather than towards health providers, has been previously proposed (7), given the growing evidence from controlled clinical trials across a wide range of different applications supporting their effectiveness.

Despite this sound rationale and research evidence supporting the use of financial incentives for patients, however, many important questions remain regarding their use, including optimal incentive values, appropriate intervention duration for different problems and populations, and cost-effectiveness. Furthermore, concerns around coercion, personal responsibility, and unintended consequences of financial incentives require further investigation, particularly for disadvantaged populations, such as those experiencing SMI or housing instability (26), who may be at greater risk of disengagement and poor health outcomes (27, 28). Despite the emergence of various models of care over the past 30 years aiming to improve access to care, engagement, and outcomes for people experiencing homelessness and mental illness, there is a paucity of research on effective strategies for engaging and retaining this population in health care settings, particularly during care transitions (29-31). Given this lack of research, and the potential role financial incentives can play in improving health and social outcomes for this population, there is a pressing need to evaluate initiatives that leverage incentives to promote engagement and continuity of care, with the potential to reduce the human and financial costs associated with homelessness and ill health.

**Study Objectives**

The present study builds on prior research by our team on interventions to improve health outcomes and reduce acute care utilization among homeless adults with mental illness discharged from acute care settings, a critical time of transition for this population. The Coordinated Access to Care for the Homeless initiative (CATCH) is a multidisciplinary brief intervention for homeless adults with mental health needs discharged from hospital in Toronto, Canada. The program was launched in 2010 and evaluated using case study methodology (32-34). CATCH bridges multiple organizations and sectors to provide coordinated physical and mental medical care, peer support and case management to homeless adults discharged from hospital for up to 6 months, and has been found to be a promising approach to improving mental health symptoms and health status, and in reducing substance misuse, and the number of hospital admissions in this population. Similar to other studies of homeless people, CATCH participants engaged with the care team variably during the study period, and maintained overall persisting high rates of acute care utilization and housing instability. The present study aims to evaluate the effect of financial incentives in facilitating treatment engagement of homeless people with mental illness, as well as in improving health, health service use and housing outcomes, compared to usual CATCH care, over 6 months, a critical time of transition from hospital to community care.

**Research Questions and Study Hypotheses**

We hypothesize that participants receiving financial incentives, compared to usual care participants, will have higher levels of service engagement (# contacts with CATCH service providers/month) over 6 months of follow up, captured by the number of appointments attended; phone calls, texts, and emails with providers; no-shows and cancellations; and drop outs from care (premature termination - before the clinicians’ recommendation). In addition, we further hypothesize that participants receiving financial incentives may also experience better health and quality of life outcomes compared to usual care participants over 6 months of follow up.

**Research Approach**

We will use a randomized controlled design and mixed methods to conduct a pragmatic field trial. Study participants will be recruited among new CATCH clients at the time of referral to CATCH and will be randomized using block randomization into receiving either the financial incentives intervention (CATCH-FI, N=86) or usual CATCH care (CATCH-UC, N = 86). This technique will maintain balanced group sizes between the CATCH-FI intervention and the CATCH-UC group at intermediate points in the recruitment process and minimize the possibility of study staff predicting group assignment. In order to operationalize a block (1:1) randomization procedure, we will create a randomization list for 176 potential participants. The list will be randomized into the Financial Incentive (FI) group or the Usual Care (UC) Group by random blocks of 4 and 6. Interviewers will assign participants to groups after completing the baseline interview. They will use sealed envelopes containing randomization assignments and will be blinded to the envelope contents prior to opening it. Interviewers will sign the envelope and take a picture of the envelope and upload on the spot. The picture will be uploaded to our servers using Dropbox.

Participant recruitment, data collection, and analysis for both the quantitative and qualitative study components are described in greater detail below.

**The Study Population**

Potential clients are referred to CATCH by hospitals or a community agency prior to or following hospital discharge. All referrals go through the program coordinator based at St. Michael’s Hospital. CATCH program eligibility criteria include 1) current homelessness (having no fixed place to stay for at least the past seven nights with little likelihood of finding a place in the upcoming month) or precarious housing (currently occupying a single room in a multi-tenant building or house with shared common areas including bathroom and kitchen or a hotel/motel as a primary residence, and having a history of one or more episodes of absolute homelessness in the past year); 2) unmet physical or mental health needs as identified by health providers; 3) unmet support needs, as identified by clients, and 4) ≥ 18 years of age. Potential clients are not admitted to the program if they demonstrate aggressive behavior requiring a higher intensity of support, or illness severity necessitating residential care.

Study participants will be recruited among successive referrals to CATCH, estimated at 450-600 referrals annually. The prevalence of severe mental illness, chronic medical conditions, alcohol, and substance use disorders of CATCH clients was found to be 86%, 75%, 35% and 49% respectively, in earlier work (32). Furthermore, among 225 CATCH participants recruited among successive program referrals, most participants were male (79%), white (65%), Canadian-born (74%), single or never married (60%), and their average age was 39.9 ± 12.0 years. Nearly all participants (88%) were documented to have at least one emergency department visit in the past 6 months, approximately half (53%) indicated at least three chronic health conditions, and 44% indicated at least three mental health diagnoses (32).

Referrals will be made by CATCH case managers (CM) during client intake meetings. The CM will briefly introduce the study to the participant using the information on the Study Handout (see Appendix D2), which they will also share with the client if requested. If the client is interested in participating, the CM will call a toll-free study phone line while they are with the client. They will reach the research team at the Survey Research Unit to set up an interview appointment, preferably on the same day. At the time of referral, research staff will verify that the client:

1. Is a new or recently (re)admitted client,
2. Has consented for the CM to share their contact information with the study team, and
3. Is available to meet for up to 2 hours to complete the interview.

For interviews that do not occur on same day as referral, the study team will use the contact information provided by the CM to follow up with participant by phone or text to remind them about their research appointment. In cases where the participant does not come to their appointment as planned, the study team will follow up with the participant to reschedule.

**Study Eligibility**

In addition to program eligibility criteria, study eligibility criteria include:

1) is a new client of the CATCH team, recently admitted or readmitted, and 2) at least one contact with the CATCH team. CATCH clients who are readmitted to hospital while receiving CATCH services are eligible to participate in the study. The baseline interview can be conducted with the participant is admitted to hospital, or soon after discharge if it is not possible to interview them while in hospital. If a client completely disengages between the time of referral to the study and the baseline interview, the 6-week window for enrollment (see page 13 for further details) restarts once they begin to engage in CATCH services again.

**Sample Size Justification**

Based on data from our previous CATCH Homeless study (32), we estimate that the number of contacts with CATCH service providers per month of program participation in the usual CATCH care group (CATCH-UC) will be equal to 4.6. We assume that participants allocated to the financial incentives (CATCH-FI) group will have at least 25% greater number of contacts, i.e., mean equal to 5.8. Because the primary outcome represents counts, sample sizes for Poisson regression are calculated using the formula provided by Signorini (35). Sample sizes of 67 per group achieve 80% power to detect a rate ratio of 1.25 with significance level of 0.05 using a two-sided test. However, based on our experience with the previous CATCH study, we expect an attrition rate of 22% and therefore the final sample size per group will be inflated to 67*(1+0.22/0.78)=86 resulting in a total of 172 participants.

**The Intervention**

CATCH clients are assigned to case managers supporting continuity and comprehensiveness of care by facilitating and coordinating their access to health and social services over 3-6 months of follow up. Case managers address immediate needs, develop comprehensive care plans with members of the multidisciplinary team (including team physicians), and support the transition of CATCH clients to longer-term community-based health and social supports. Clients also have access to a weekly clinic with physicians and a nurse at the Good Shepherd Centre.

Successful engagement and program participation is instrumental in achieving continuous comprehensive care that is tailored to the needs of homeless people with mental health needs. We will examine the effect of providing financial incentives of $20 to clients in the CATCH-FI cohort for every week they maintain contact with CATCH service providers, as required by their care plan. Contact can be by phone, text, email, or in person with CATCH service providers over 6 months of follow up, or until they are successfully transitioned to longer-term supports (for up to $80/month per participant). Financial participants who complete the 6-month interview early in the 22-week data collection window (further described under *Data Collection*) will continue to be eligible to earn the weekly $20 financial incentive until exactly 6 months after the date of their baseline interview. This will be explained to participants during the 6-month interview and they will have the opportunity to collect their incentive at a set weekly distribution time. A participant will be considered to have completed a contact if they have a phone call or text message conversation lasting 5 minutes or longer, or a face to face contact, as appropriate. This definition is consistent with the current practice of the CATCH team, to ensure only meaningful contacts are recorded and reported to their funder. The effect of financial incentives on engagement, as well as health, acute care utilization and housing outcomes will be examined over 6 months of follow up. The primary outcome of effectiveness will be the number of contacts (per month) with CATCH service providers (established by review of program records) until the transition to long-term care providers is accomplished. Secondary outcomes will include: (i) number of hospitalizations, days in hospital, and the number of emergency department visits over 6 months, established through both self-report and administrative databases at ICES, and (ii) changes in health status, mental health, substance use and quality of life, using validated measures extensively used by our team in prior research with this population.

At the time of consent to the study, all participants will be reminded of the benefits to their care coordination of ongoing contact with CATCH providers. Surveys will be administered at baseline and 6 months to all study participants, using the following measures, described in detail on page 14:

1. Demographic information, including age, gender, race, education level and housing history
2. All sources of income in the past 30 days for self and partner (if applicable)
3. Quality of life, using QoLi-20
4. Substance use, using the GAIN-SS
5. Working alliance, using the WAI-SR
6. Mental health symptom severity, using the CSI
7. Health status, using the SF-36
8. Health-related quality of life, using the EQ-5D-5L
9. Housing stability, using the RTLFB
10. Self-reported number of ED visits, hospital admissions and days in hospital in the past 6 months and
11. Health care service use over 2 years, using various Ontario health databases

To achieve an in-depth understanding of both the acceptability, and risks and benefits of financial incentives, we will also undertake a qualitative study engaging service users, providers and other key informants in individual interviews and focus groups exploring the acceptability and impact of financial incentives, as well as factors contributing to health decision making and treatment engagement in this population. The study is seeking approval from the Research Ethics Board of Unity Health Toronto and CAMH.

**Methodology**

*Quantitative methods*

The primary research question is: Are financial incentives effective in improving treatment engagement and health outcomes, compared to usual care, for homeless people with mental health needs 6 months after enrolment in a brief intervention? The primary measure of effectiveness is treatment engagement, measured as a number of contacts per month with CATCH service providers over the period the participant is in the program. The secondary measures of effectiveness are changes from baseline to 6 month follow-up in 1) health status; acute care service use; 2) days stably housed; 3) mental health; 4) substance use, and 5) quality of life.

*Qualitative Evaluation*

The research question for the qualitative component is: What are the role and the advantages and disadvantages of financial incentives in supporting engagement with care pathways for homeless people with mental health needs from the perspective of service users providers, and community stakeholders? The qualitative study component will include interviews and focus groups with CATCH clients from both the FI and UC groups, CATCH service providers, and key informant stakeholders and other community agencies. This will provide in-depth understanding of factors affecting health decision making and treatment engagement in this population, as well as an exploration of the acceptability and impact of financial incentives for this population.

*Capacity to provide informed consent*

We anticipate that most participants will be able to understand and participate fully in the consent process. However, to confirm capacity to consent, a capacity to consent questionnaire will be used to confirm participants’ understanding of key aspects of the letter of information and consent from prior to signing. Additionally, during recruitment and interview scheduling, we will offer access to a professional interpreter for anyone having difficulty communicating in English. In these cases, the interpreter will be asked to sign the ‘Interpretation Declaration’ section of the consent form.

**Data Collection**

*Quantitative Data*

The study will utilize data collection instruments and knowledge gained from the original CATCH and At Home / Chez Soi studies to successfully recruit and retain this population.

Baseline and 6-month follow up surveys will be completed by trained research assistants (RA) from the Survey Research Unit at the Centre for Urban Health Solutions at St. Michael’s Hospital and will last approximately 1-2 hours. The RAs, supported by a Research Coordinator (RC), will be responsible for recruiting study participants, as well as extracting clinical record data to ascertain the frequency of contact with CATCH clinicians, (number of contacts, no-shows, service duration). To minimize study attrition, at the baseline interview, study participants will be asked to provide detailed contact information, as well as names and phone numbers of family, acquaintances and other service providers that can be contacted (with participant consent) if the research team cannot reach the participant. Finally, to further support participant retention over the follow-up period, study participants will be encouraged to call study staff monthly after their baseline interview to update the name and phone numbers for contact and will be provided with a $10 honorarium for each of such calls. If participants do not call to update or confirm their information, study staff will use their previously provided contact information to occasionally reach out to participants between interviews to maintain up to date contact records. These strategies have facilitated high follow up rates with this population and have been implemented successfully by others (36).

All baseline interviews will be completed as soon as possible, within the first 6-weeks of program enrollment. A data collection window of 22 weeks (up to six weeks’ prior to or sixteen weeks after the 6-month time point) will be allowed for the 6-month visits. Study participants will receive an honorarium of $30 at baseline and $60 at follow up, and public transportation fares for each interview. Finally, the research team will check program records to establish the number of weekly contacts with CATCH service providers and provide participants in the CATCH-FI group an additional $20 for every week they maintain contact (for up to $80/month per participant). For telephone interviews, participants can choose to receive this honorarium as a mailed cheque to their preferred address or as an email money transfer. Participants can also elect to receive any owed monthly check-in payments or financial incentive payments through the above noted payment methods.

The study team will be using electronic based surveys using SNAP Professional software for data collection.  Please note that SNAP Professional Software has been reviewed and approved for use by St. Michael Hospital. The Snap server utilized is owned by the Survey Research Unit and is located inside the St. Michael’s Hospital network.

A summary of survey instruments and outcome variables is provided below:

| Domain and *Selected Instrument* | Description of Domain and/or Instrument |
| --- | --- |
| Domain: Socio-demographic variables  Instrument: *Self-report* | Research staff will collect the following information at baseline only: age, gender, race, country of birth, main language.  Information collected at baseline and 6-months includes: education level, marital status, residential status (duration of homelessness during their lifetime and during the past 6 months). |
| Domain: Income  Instrument: *Self-report* | Income will be collected for participant and partner (if applicable) from the following sources: all jobs, Ontario Works, Ontario Disability Support Program, Employment Insurance, Child Benefits, Child Support, and all other sources of income. |
| Domain:  Disease-Specific Quality of Life  Instrument:  *Quality of Life Index-20 (QoLi-20)* | The original Lehman Quality of Life scale was designed to assess the quality of life of people with severe mental illness. It is a structured self-report interview, conducted by a trained non-clinical interviewer, and elicits participants’ ratings of their quality of life. There are 7 subjective scales (living situation, everyday activities, family, social relationships, finances, safety, and satisfaction with life in general) and 4 objective scales (everyday activities, enough money, family contacts, and contacts with friends). This shorter 20-item version was developed and validated by Uttaro and Lehman (37) and used extensively in the homeless population. |
| Domain: Substance use  Instrument: *Global Appraisal of Individual Needs Short Screener* | The GAIN-SS, extensively used in this population, consists of questions to determine participants’ severity of substance use problems (such as getting into fights, problems at work, dealing with withdrawal symptoms) in the ‘past month’, ‘2–3 Months’, ‘4–12 Months’ or ‘1+ years.’ Using these questions, the GAIN past month score is calculated by counting the number of times the participant identified that they had these problems in the last month. In addition, we will elicit information on the number of days in the 30 days that participants have problems with alcohol or drugs, and the amount of money spent on alcohol or drugs in the past 30 days. |
| Domain: Working Alliance  Instrument: *Working Alliance Inventory – Short Revised* | Participants will be asked to complete the 12-item Working Alliance Inventory - Short Revised (WAI-SR) questionnaire, to assess how they think and feel about the therapeutic relationship with their case manager. Responses are provided using a 5 point Likert scale ranging from 1 (Seldom) to 5 (Always).The WAI-SR includes three subscales relating to task, goal, and bond, and yields a summary score that ranges from 12 to 60, with higher scores indicating a stronger therapeutic relationship. |
| Domain: Mental health symptom severity  Instrument: *Colorado Symptom Index* | The CSI was designed specifically for homeless individuals with mental health problems. It is a 14-item instrument which assesses the presence and frequency of symptoms of mental illness experienced within the past month. Responses are provided using a 5-point Likert scale with answer choices ranging from 0 (not at all) to 4 (at least every day). A higher score indicates a higher level of symptoms. The Colorado Symptom Index is a widely used tool in research as a self-report measure of psychiatric symptomatology. The CSI has been reported to have excellent internal consistency (.92) and test-retest reliability (.71). Evidence of the CSI’s validity is strong as CSI scores have been proven to distinguish between individuals with and without mental health service needs and were significantly correlated with functioning (43). |
| Domain:  Health Status  Instrument:  *Short Form 36* | This 36-item self-report measure of generic health status is designed to produce Physical Component Summary (PCS) and Mental Component Summary (MCS) scores. There are questions concerning physical functioning, role limitations, bodily pain, general health perceptions, vitality, social functioning, and general mental health (psychological distress and psychological well-being). SF-36 has excellent psychometric properties and has been used successfully in a variety of settings and diagnostic groups, including the homeless population (44). |
| Domain: Health-related quality of life  Instrument: *EQ-5D-5L* | The EQ-5D-5L is a generic measure of health-related quality of life. The EQ-5D-5L includes five items concerning mobility, self-care, usual activities, pain/discomfort, and anxiety/depression that are weighted to produce a single utility score between 0 and 1. The Visual Analog Scale (VAS) of the EQ-5D-5L will also be included, which will allow participants to rate their overall health, mental health and physical health from 0 to 100. |
| Domain: Housing Stability  Instrument:  *Dartmouth Residential Time-Line Follow-Back* | This modified version of the RTFLB is designed to collect detailed information about a participant’s type of housing and number of days stably housed. The RTFLB uses a calendar and prompts to collect housing for specific time periods. In this study, this information will inform questions about housing stability and circumstances and is being collected at baseline and 6 months (49, 50). The outcome of interest is the number of days stably housed in the past 6 months. |
| Domain: Acute Care Utilization  Participant Service Use  Instrument: *Self-report and* *Administrative Data* | Service use events will be tracked using both self-reported and administrative data. Regarding self-reported data, we will establish the number of hospitalizations, days in the hospital and emergency room visits during the 6 months prior to program enrolment as well as the 6 months follow up period. |
| Domain: Health Service Use  Instrument: *Administrative Data* | We will establish the number of hospitalizations, days in hospital and emergency department visits during the year prior to program enrolment and the 1 year follow-up period, for a total of 2 years. Data linkage will be conducted at the Institute for Clinical Evaluative Sciences (ICES), where population-based health information is available at the patient level for all Ontarians using formal health services. Health service use will be examined by the National Ambulatory Reporting System (NACRS), the Discharge Abstract Database (DAD), the Ontario Mental Health Reporting System (OMHRS) and the Ontario Health Insurance Plan (OHIP) for ED visits, outpatient clinics, and inpatient hospitalizations. The project will attain health service data for all consenting participants from 12 months prior to study enrolment to 12 months following study enrolment. |

*Qualitative Data*

Participant Recruitment and Data Collection

Interviews and focus groups will be conducted by research staff from the Survey Research Unit at the Centre for Urban Health Solutions at St. Michael’s Hospital trained in qualitative methods. The research team will meet regularly with the Principal Investigator prior to and throughout data collection to refine the interview guides and prompts using an iterative process in attempts to better gauge participant perspectives (51). Consistency between interviews will be ensured by interviewer training and review of early transcripts by the research team and the PI. All interviews and focus groups will be audio-recorded and transcribed verbatim.

In-depth, 45-60 minute semi-structured interviews will be conducted with 15-22 client participants and 2-6 key informants including policy and decision makers. Furthermore, 2-3 semi-structured 1-1.5 hour focus groups with approximately 12 additional stakeholders will be conducted, including CATCH service providers and external service providers servicing the target population. Client participants will be purposefully recruited among those already enrolled in the quantitative study who are able to reflect on their experiences, and represent a diversity of perspectives in terms of gender, age, race, and study arm participation. Client participants will be compensated $30 and public transportation fares for their time. The interview and focus group guides will solicit information on perceptions of barriers and facilitators to service engagement during transitions of care, factors affecting health decision making in this population, as well the perceived acceptability, risks, barriers, and expected or experienced impact of financial incentives during care transitions.

Data collection for quantitative and qualitative measures will take place between November 2018 and October 2020 in Toronto, Canada. Please see Appendices C1-C3 for draft interview and focus group guides.

**Quantitative Data Analysis**

Exploratory analyses will calculate descriptive statistics (mean, standard deviation, median, quartiles), construct graphs (histograms, box-plots, scatterplots, spaghetti plots), and estimate correlations between selected participants’ characteristics and longitudinal outcomes.

*Primary Outcome Analysis*

Since program duration is customized for each participant, and may last between 1 and 6 months, we will calculate participants’ person-months in order to estimate the rate ratio comparing CATCH-FI with CATCH-UC with respect to the number of contacts with CATCH service providers per month. Therefore, for each participant, the total number of months in the program before discharge and the total number of contacts over the number of months in the program will be calculated. A Poisson regression model (PROC GENMOD) with total contacts as the dependent variable, group (CATCH-FI vs CATCH-UC) as the covariate and an offset equal to the log (number of months spent on the program) will estimate the rate ratio and 95% confidence intervals, and the mean number of contacts per person-months and 95% confidence intervals in each group.

*Secondary Outcome Analysis*

For continuous outcomes (i.e., QoLi-20, CSI, SF-36 and EQ-5D VAS), we will define change from baseline to 6 months follow-up as scores at 6 months minus scores at baseline. We will conduct analysis of covariance (ANCOVA) to compare change from baseline between CATCH-FI and CATCH-UC adjusting for baseline scores as a covariate.

For count outcomes (i.e., GAIN-SS, number of hospitalizations, number of days hospitalized, and number of emergency department visits) we will model the baseline and 6 months outcomes using generalized estimating equations (GEE) assuming the Poisson distribution or the negative binomial distribution if over-dispersion is suggested by the data. The models will include the main effects of group (CATCH-FI vs CATCH-UC) and time (6 months vs. baseline), and the interaction of group by time. A significant interaction will indicate that change from baseline is different between the groups. Rate ratios and 95% confidence intervals will be estimated.

The analysis of administrative data is similar to that of count outcomes, except that the period of consideration will be 12 months instead of 6 months pre and post-randomization.

For analyzing the number of days stably housed in the past 6 months, we will consider generalized estimating equations with a Poisson or negative binomial distribution. The model will include the main effects of group and time, an interaction between group and time, selected covariates, and an offset represented by the natural log of residence days accounted during the 6 months interval. Rate ratios and 95% confidence intervals will be estimated to compare the rate of days stably housed per person-months.

For the WAI-SR, evaluated at 6 months follow-up, total scale and sub-scales scores will be calculated and compared between the groups using the two-sample t-test or the Wilcoxon rank-sum test if extreme outliers are present. The correlation between WAI-SR and other outcomes at 6 months will be explored by estimating the Pearson of Spearman correlation coefficients, overall and by group.

SAS 9.4 will be used for all analyses. All statistical tests will be two-sided and a p-value of 0.05 or less will indicate statistical significance.

**Qualitative Analysis**

*Interviews and Focus Groups*

Qualitative interview and focus group data will be analyzed through an inductive thematic analysis approach using NVivo software (52). A team of coders comprised of research staff and investigators will review transcripts and develop a set of key concepts or ‘codes’ (53). Through a close reading of each source, relevant excerpts will be assigned to one or more codes. Some codes may be identified beforehand based on literature reviews and initial impressions of the data, while others will emerge during the coding phase. Once all data is coded, similar codes will be grouped into a set of high-level themes, supported with direct examples and quotations from the sources. At this stage, the research team will discuss the categories and collectively reduce them to a smaller set of higher-level themes (54). The same inductive analysis approach will be used in examining program documents if any.

**Data retention**

The study personnel will make every effort to keep personal health information private and confidential in accordance with all applicable privacy legislation, including the Personal Health Information Protection Act (PHIPA) of Ontario. Any health information that is recorded for study purposes will be de-identified by using a unique study identification number instead of any identifying information and stored in a Master Linking Log. This information will only be used to get in touch with participants and access health records with participant consent. It will only be seen by study staff who are not connected to any part of participant health care, and will stay at St. Michael’s Hospital’s secure computer server in a password protected file. The principal investigator at St. Michael’s Hospital is in control of the Master Linking Log.

At each interview, surveys will be collected using either a paper version or electronic web version of SNAP Professional Software. All of the paper data will be stored in locked filing cabinets that only authorized members of the research team will have access to. All of the electronic data will be kept on a secure server at St. Michael’s Hospital in an unreadable format for anyone outside of the study. Only authorized members of the research team will have access to the survey data. All study information will be kept for a period of 10 years from the end of the study and then destroyed. The Principal Investigator will protect participant records and keep all information confidential to the greatest extent possible by law.

Research staff may use texting and email to set up appointments with participants, if they indicated on their Contact Information Sheet that they wished to be contacted in this way. There is no obligation to text or email – participants may always contact the research team by phone or through their CATCH case manager. The research team will not collect any participant personal health information through email or texting. In the consent form, participants are advised that email and text messages are not secure modes of communication, and asked not to send any personal health information via text or email, and not to use text or email in emergency situations. Research staff will only use a participant’s first name in a text message. If the research team does receive personal health information or a notice of an emergency by text or email, the research team will follow up with a phone call. Text messages will be stored on a password protected study cell phone held by the Survey Research Unit at St. Michael’s Hospital, and will be seen, sent, and retained by the research team. Emails will be hosted on St. Michael’s hospital Outlook email server, and also seen, sent, and retained by the research team. Appointment information discussed by text or email will be entered into the participant file. Once this is completed, the texts and emails will be deleted.

Participants will be asked if they are interested in being contacted regarding additional related research for three years after the completion of the study. If so, participant contact information will be maintained for this time period. If not, it will be destroyed after study completion.

**Study Limitations**

Several limitations of the proposed study can be noted. First, the study is taking place in a single urban center in Ontario and may not capture the diversity of service delivery contexts facing similar challenges in supporting care transitions for people experiencing homelessness. Thus, findings from this initial study should serve as a springboard for a larger, multisite randomized controlled trial. Secondly, every intervention involving financial incentives faces the limitation of creating a differential effect on those with varying levels of financial need. Given however that this study is focused exclusively on homeless people, this risk is minimal. Third, indices of service engagement are based on process measures, serving as proxy measures to service engagement. This approach has been used extensively in the psychotherapy literature, however, and in the absence of validated tools of treatment engagement, it can offer valuable information. To further understand service engagement, we will also measure the working alliance between clients and providers at 6 months.

**Research Team**

The research team is led by Dr. Vicky Stergiopoulos and includes Dr. Stephen Hwang, Dr. Rosane Nisenbaum, Dr. Nicole Kozloff and Dr. Anna Durbin.

Vicky Stergiopoulos, MSc, MD, MHSc, is a clinician-scientist, an Associate Scientist at the Centre for Urban Health Solutions at St. Michael’s Hospital, the Physician-in-Chief at the Centre for Addiction and Mental Health, and Professor and Vice Chair, Clinical and Innovation in the Department of Psychiatry at the University of Toronto. Her expertise includes mental health services research, including the design, implementation, and evaluation of interventions for people experiencing homelessness using both qualitative and quantitative methods. Dr. Stephen Hwang, MD, MPH, is a clinician-scientist, the Director of the Centre for Urban Health Solutions at St. Michael’s Hospital, and a Professor of Medicine at the University of Toronto. His research has focused on homelessness and health services research. Rosane Nisenbaum, PhD, is a Senior Biostatistician at the Centre for Urban Health Solutions, an Assistant Professor at the Dalla Lana School of Public Health, with extensive experience analyzing datasets inclusive of the instruments utilized in this study. Dr. Anna Durbin is a scientist at the Centre of Urban Health Solutions and Assistant Professor in the Department of Psychiatry at the University of Toronto, with expertise in survey and administrative data analysis. Dr. Nicole Kozloff is a clinician scientist at the Centre for Addiction and Mental Health and an Assistant Professor in the Department of Psychiatry at the University of Toronto. She has expertise in mental health services research using both qualitative and quantitative methodologies.

**Research Staff**

The Survey Research Unit at the Centre for Urban Health Solutions, St. Michael’s Hospital, will provide research staff for survey database development, study recruitment, and data collection for both quantitative and qualitative components. Dr. Tatiana Aratangy, the Unit Lead, and Cheryl Pedersen, a Research Manager in the SRU, will oversee the project. Dr. Aratangy and Cheryl have expertise in primary data collection, research design and program evaluation. Under their direction, the Survey Research Unit has successfully completed multiple projects involving people experiencing homelessness and SMI. Rebecca Brown is a Research Coordinator with the Survey Research Unit. Under Dr. Aratangy’s and Cheryl’s direction, Rebecca will be the lead coordinator on this project. Rebecca has experience in both quantitative and qualitative data collection as well as experience coordinating multi-site and mixed-methods projects. Kate Francombe Pridham is a Research Coordinator coordinating all studies at C-UHS funded through the Health System Research Fund, including this project. Kate has experience at St. Michael’s with quantitative and qualitative project management, data collection and analysis, focusing on studies on mental health and homelessness.

**Risks and Benefits**

*Risks*

Involvement in this research poses no to minimal risks to participants. There is a possibility that some participants may find certain survey or interview questions to be challenging or uncomfortable. However, participation is entirely voluntary and individuals may choose to not answer specific questions or withdraw from the study at any point in time without penalty. Should participants request additional supports, they will be provided with a list of available resources. Should an individual choose to withdraw from the study entirely, they can request that all information collected from them to that point be destroyed. The Principal Investigators bring extensive experience in the design, implementation, and evaluation of interventions for the target population, providing an excellent foundation for early identification and prompt response to potential emerging challenges.

*Benefits*

There are no direct benefits for study participants in the CATCH-FI group. There may be indirect benefits to all study participants from sharing their experiences with the research team. Study findings will be used to improve CATCH services and inform the wider use of financial incentives for this population in Ontario and beyond.

References

1. Bosworth HB, Granger BB, Mendys P, Brindis R, Burkholder R, Czajkowski SM, et al. Medication Adherence: A Call for Action. American heart journal. 2011;162(3):412-24.

2. Haddad PM, Brain C, Scott J. Nonadherence with antipsychotic medication in schizophrenia: challenges and management strategies. Patient Related Outcome Measures. 2014;5:43.

3. Gutnik LA, Hakimzada AF, Yoskowitz NA, Patel VL. The role of emotion in decision-making: A cognitive neuroeconomic approach towards understanding sexual risk behavior. Journal of Biomedical Informatics. 2006;39(6):720-36.

4. Operario D, Kuo CC, Sosa-Rubí SG, Gálarraga O. Conditional Economic Incentives for Reducing HIV Risk Behaviors: Integration of Psychology and Behavioral Economics. Health psychology : official journal of the Division of Health Psychology, American Psychological Association. 2013;32(9):932-40.

5. Giles EL, Robalino S, McColl E, Sniehotta FF, Adams J. The Effectiveness of Financial Incentives for Health Behaviour Change: Systematic Review and Meta-Analysis. PLoS ONE. 2014;9(3):e90347.

6. Lagarde M, Haines A, Palmer N. Conditional cash transfers for improving uptake of health interventions in low- and middle-income countries: A systematic review. JAMA. 2007;298(16):1900-10.

7. Wu J. Rewarding Healthy Behaviors—Pay Patients for Performance. Annals of Family Medicine. 2012;10(3):261-3.

8. Farber S, Tate J, Frank C, Ardito D, Kozal M, Justice AC, et al. A Study of Financial Incentives to Reduce Plasma HIV RNA Among Patients in Care. AIDS and Behavior. 2013;17(7):2293-300.

9. Bassett IV, Wilson D, Taaffe J, Freedberg KA. Financial incentives to improve progression through the HIV treatment cascade. Curr Opin HIV AIDS. 2015;10(6):451-63.

10. Tappin D, Bauld L, Purves D, Boyd K, Sinclair L, MacAskill S, et al. Financial incentives for smoking cessation in pregnancy: randomised controlled trial. Bmj. 2015;350:h134.

11. Banerjee AV, Duflo E, Glennerster R, Kothari D. Improving immunisation coverage in rural India: clustered randomised controlled evaluation of immunisation campaigns with and without incentives. The BMJ. 2010;340:c2220.

12. Volpp KG, Troxel AB, Pauly MV, Glick HA, Puig A, Asch DA, et al. A randomized, controlled trial of financial incentives for smoking cessation. The New England journal of medicine. 2009;360(7):699-709.

13. Volpp KG, John LK, Troxel AB, Norton L, Fassbender J, Loewenstein G. Financial incentive-based approaches for weight loss: a randomized trial. Jama. 2008;300(22):2631-7.

14. Merrick EL, Hodgkin D, Horgan CM. Incentives to Shape Health Behaviors: How Can We Make Them More Person-Centered? J Workplace Behav He. 2014;29(1):19-31.

15. Stanley IH, Chu C, Brown TA, Sawyer KA, Joiner TE, Jr. Improved Clinical Functioning for Patients Receiving Fee Discounts That Reward Treatment Engagement. Journal of clinical psychology. 2016;72(1):15-21.

16. Post E, Cruz M, Harman J. Incentive Payments for Attendance at Appointments for Depression Among Low-Income African Americans. Psychiatric Services. 2006;57(3):414-6.

17. Gupta S. CONTINGENCY MANAGEMENT Why it pays to quit. Nature. 2015;522(7557):S57-S9.

18. Messina N, Farabee D, Rawson R. Treatment responsivity of cocaine-dependent patients with antisocial personality disorder to cognitive-behavioral and contingency management interventions. Journal of consulting and clinical psychology. 2003;71(2):320.

19. Lussier JP, Heil SH, Mongeon JA, Badger GJ, Higgins ST. A meta-analysis of voucher-based reinforcement therapy for substance use disorders. Addiction (Abingdon, England). 2006;101(2):192-203.

20. Prendergast M, Podus D, Finney J, Greenwell L, Roll J. Contingency management for treatment of substance use disorders: a meta-analysis. Addiction (Abingdon, England). 2006;101(11):1546-60.

21. Burton A, Marougka S, Priebe S. Do financial incentives increase treatment adherence in people with severe mental illness? A systematic review. Epidemiologia e psichiatria sociale. 2010;19(3):233-42.

22. Priebe S, Burton A, Ashby D, Ashcroft R, Burns T, David A, et al. Financial incentives to improve adherence to anti-psychotic maintenance medication in non-adherent patients - a cluster randomised controlled trial (FIAT). BMC Psychiatry. 2009;9:61.

23. Priebe S, Yeeles K, Bremner S, Lauber C, Eldridge S, Ashby D, et al. Effectiveness of financial incentives to improve adherence to maintenance treatment with antipsychotics: cluster randomised controlled trial. Bmj. 2013;347:f5847.

24. Priebe S, Bremner SA, Pavlickova H. Discontinuing financial incentives for adherence to antipsychotic depot medication: long-term outcomes of a cluster randomised controlled trial. BMJ Open. 2016;6(9):e011673.

25. Serumaga B, Ross-Degnan D, Avery AJ, Elliott RA, Majumdar SR, Zhang F, et al. Effect of pay for performance on the management and outcomes of hypertension in the United Kingdom: interrupted time series study. BMJ. 2011;342.

26. Meaden A, Hacker D, de Villiers A, Carbourne J, Paget A. Developing a measurement of engagement: the Residential Rehabilitation Engagement Scale for psychosis. Journal of mental health (Abingdon, England). 2012;21(2):182-91.

27. Priebe S, Sinclair J, Burton A, Marougka S, Larsen J, Firn M, et al. Acceptability of offering financial incentives to achieve medication adherence in patients with severe mental illness: a focus group study. Journal of Medical Ethics. 2010;36(8):463-8.

28. Black C, Gronda H. Evidence for improving access to homelessness services: Australian Housing and Urban Research Institute; 2011.

29. Smith TE, Burgos J, Dexter V, Norcott J, Pappas SV, Shuman E, et al. Best practices: Best practices for improving engagement of clients in clinic care. Psychiatric services (Washington, DC). 2010;61(4):343-5.

30. Tait L, Birchwood M, Trower P. A new scale (SES) to measure engagement with community mental health services. Journal of mental health (Abingdon, England). 2002;11(2):191-8.

31. Kotwicki RJ, Balzer AM, Harvey PD. Measuring and Facilitating Client Engagement with Financial Incentives: Implications for Improving Clinical Outcomes in a Mental Health Setting. Community Ment Health J. 2017;53(5):501-9.

32. Stergiopoulos V, Gozdzik A, Nisenbaum R, Lamanna D, Hwang SW, Tepper J, et al. Integrating Hospital and Community Care for Homeless People with Unmet Mental Health Needs: Program Rationale, Study Protocol and Sample Description of a Brief Multidisciplinary Case Management Intervention. International Journal of Mental Health and Addiction. 2017;15(2):362-78.

33. Lamanna D, Stergiopoulos V, Durbin J, O'campo P, Poremski D, Tepper J. Promoting continuity of care for homeless adults with unmet health needs: The role of brief interventions. Health & social care in the community. 2018;26(1):56-64.

34. Stergiopoulos V, Gozdzik A, Tan de Bibiana J, Guimond T, Hwang SW, Wasylenki DA, et al. Brief case management versus usual care for frequent users of emergency departments: the Coordinated Access to Care from Hospital Emergency Departments (CATCH-ED) randomized controlled trial. BMC Health Services Research. 2016;16(1):432.

35. Signorini, D. F. (1991). Sample size for Poisson regression. Biometrika, 78(2), 446-450.

36. Larry J. Seidman, Russell K. Schutt, Brina Caplan, George S. Tolomiczenko, Winston M. Turner, Stephen M. Goldfinger. The Effect of Housing Interventions on Neuropsychological Functioning Among Homeless Persons With Mental Illness. Psychiatric Services. 2003;54(6):905-8.

37. Uttaro T, Lehman, A. Graded response modeling of the Quality of Life Interview. Evaluation and Program Planning. 1999;22:41-52.

38. Horvath AO, Greenberg LS. The working alliance: Theory, research, and practice: John Wiley & Sons; 1994.

39. Busseri MA, Tyler JD. Interchangeability of the working alliance inventory and working alliance inventory, short form. Psychological assessment. 2003;15(2):193.

40. Horvath AO, Greenberg LS. Development and validation of the Working Alliance Inventory. Journal of counseling psychology. 1989;36(2):223.

41. Tracey TJ, Kokotovic AM. Factor structure of the working alliance inventory. Psychological Assessment: A journal of consulting and clinical psychology. 1989;1(3):207.

42. Tichenor V, Hill CE. A comparison of six measures of working alliance. Psychotherapy: Theory, Research, Practice, Training. 1989;26(2):195.

43. Boothroyd RA, Chen HJ. The psychometric properties of the Colorado Symptom Index. Administration and policy in mental health. 2008;35(5):370-8.

44. Lehman AF, Dixon LB, Kernan E, DeForge BR, Postrado LT. A randomized trial of assertive community treatment for homeless persons with severe mental illness. Archives of general psychiatry. 1997;54(11):1038-43.

45. Lamers LM, Bouwmans CA, van Straten A, Donker MC, Hakkaart L. Comparison of EQ-5D and SF-6D utilities in mental health patients. Health economics. 2006;15(11):1229-36.

46. EuroQol--a new facility for the measurement of health-related quality of life. Health policy (Amsterdam, Netherlands). 1990;16(3):199-208.

47. Brooks R. EuroQol: the current state of play. Health policy (Amsterdam, Netherlands). 1996;37(1):53-72.

48. Rasanen P, Roine E, Sintonen H, Semberg-Konttinen V, Ryynanen OP, Roine R. Use of quality-adjusted life years for the estimation of effectiveness of health care: A systematic literature review. International journal of technology assessment in health care. 2006;22(2):235-41.

49. Tsemberis SM, G., Williams, V., Hanrahan, P. and Stefancic, A. . Measuring homelessness and residential stability: The Residential Time-Line Follow-Back Inventory. Journal of Community Psychology. 2007;35(1):29-42.

50. Center NHDR. Residential Follow-Back Calendar [version June 1995]. Lebanon, NH: Dartmouth Medical School; 1995.

51. Hashimov E. Qualitative Data Analysis: A Methods Sourcebook and The Coding Manual for Qualitative Researchers: Matthew B. Miles, A. Michael Huberman, and Johnny Saldaña. Thousand Oaks, CA: SAGE, 2014. 381 pp. Johnny Saldaña. Thousand Oaks, CA: SAGE, 2013. 303 pp. Taylor & Francis; 2015.

52. Buchanan J, Jones ML. The efficacy of utilising Nvivo for interview data from the electronic gaming industry in two jurisdictions. 2010.

53. Saldaña J. The coding manual for qualitative researchers: Sage; 2015.

54. Berg B. Qualitative Research Methods for the Social Sciences. 7th ed. Boston Allyn & Bacon; 2008.
